# The Social Relations and Economic Preparation for Retirement of One-Person Households

**DOI:** 10.1093/geroni/igab046.3291

**Published:** 2021-12-17

**Authors:** Heayoung Sung

**Affiliations:** National Pension Research Institute, Jeon-Ju si, Jeollabuk-do, Cholla-bukto, Republic of Korea

## Abstract

Objectives: The purpose of this study is to find out the effect of middle-aged one-person households on social relations and economic preparation for retirement. In addition, even within one-person households, I attempted to determine the difference according to whether the cause of the households’ composition was separation after marriage or a one-person household without a spouse due to being unmarried, bereavement, or divorce. Methods: As a result of the analysis using the data of the 2019 Korean Social Survey of the National Statistical Office, single-person households had weaker economic conditions, social relations, and economic preparation for retirement when compared to multi-person households. One-person households lacked a social network to receive help when they face difficulties compared to multi-person households, but there was no effect on group activities or the satisfaction with human relationships. However, households with no spouse had a negative impact on the social network, group activities, and the satisfaction with human relationships. Conclusions: Middle-aged one-person households and one-person households with no spouse had a negative effect on economic preparation for retirement, when labor activity and asset variables were not considered. However, statistical significance was not found when these variables were considered.

